# Preselector.uni-jena.de: optimize your cloning—a resource for identifying restriction enzymes for preselection reactions

**DOI:** 10.1093/nar/gkab406

**Published:** 2021-05-25

**Authors:** Martin Gühmann, Stefanie Reuter, Jan Hartung, Ralf Mrowka

**Affiliations:** University of Bristol, School of Biological Sciences, 24 Tyndall Avenue, Bristol BS8 1TQ, UK; ThIMEDOP, Universitätsklinikum Jena, Am Nonnenplan 4, D-07743 Jena, Germany; Experimentelle Nephrologie, Universitätsklinikum Jena KIM III, Am Nonnenplan 4, D-07743 Jena, Germany; Experimentelle Nephrologie, Universitätsklinikum Jena KIM III, Am Nonnenplan 4, D-07743 Jena, Germany

## Abstract

Preselection digests are a common strategy to reduce the background in the ligation step of molecular cloning. However, choosing fitting restriction enzymes by hand is not trivial and may lead to errors, potentially costing a lot of time and work. We therefore created preselector.uni-jena.de (https://preselector.uni-jena.de/), a free online tool to find such restriction enzymes. The tool uses regular expressions to find recognition sites of restriction enzymes in the DNA sequences provided by the user. This new tool compares the sets of restriction sites and reports the enzymes that cut one sequence but not the other sequences to the user. These enzymes are then the ones suitable for a preselection digest. Thus, preselector.uni-jena.de is a fast, reliable, and free-to-use tool to help researchers designing preselection digestion strategies for their cloning.

## Graphical Abstract

**Graphical Abstract:**
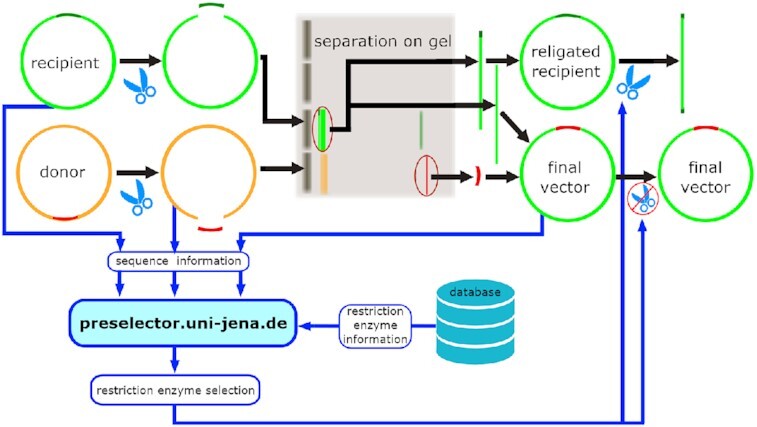
Find your preselection enzymes with preselector.uni-jena.de.

## INTRODUCTION

**Figure 1.**
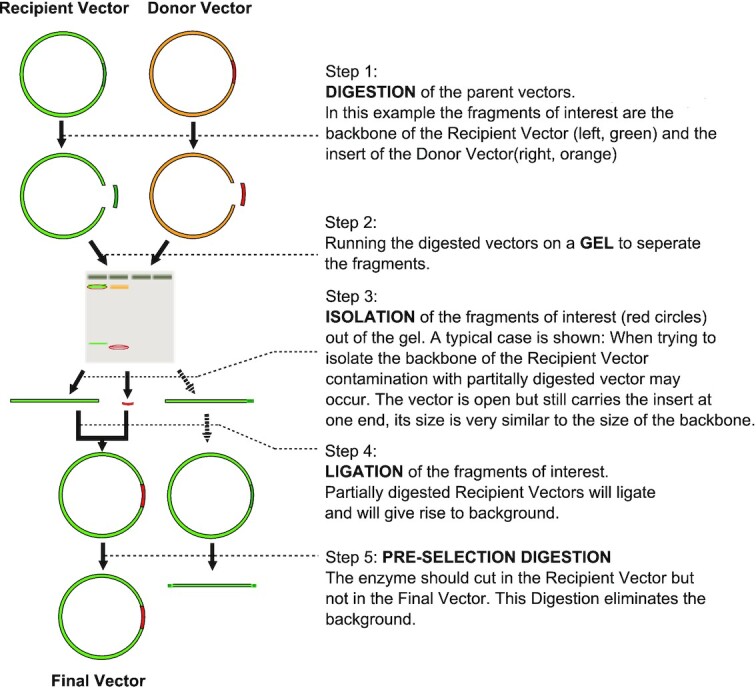
A typical approach in molecular cloning with preselection. Preselection digests allow to remove those plasmids not wanted for transformation. The plasmids are treated with an enzyme that cuts the recipient plasmid but not the final plasmid. This way, the unwanted plasmids become linearized and are unsuitable for transformation since they do not replicate in E. coli.

Molecular cloning is a basic method in molecular biology and has many applications such as genome editing with CRISPR–Cas9 or the development and production of nucleotide-based vaccines. Here, restriction enzymes are used to cut out a fragment of DNA from a donor plasmid to ligate it afterwards into a previously opened recipient vector (1). Typically, the DNA fragments are separated on and purified from an agarose gel before ligation (Figure 1), which is a source for contamination. Molecular cloning allows combining DNA fragments from different sources into one circular double stranded DNA molecule.

Depending on the enzyme and on the reaction conditions, the digestion reaction may be incomplete, and this is in some cases hard to detect. This is a problem if the vector backbone has several thousands of base pairs and the cutout fragment is considerably shorter, e.g. just 100 bp. In this case the fragments can hardly be separated, and thus the following ligation reaction is contaminated with partially digested recipient vectors. Even if the fraction of partially digested vectors is low, partially digested vectors can easily religate compared to multiple fragments that have to come in proximity first. This, in addition to contamination with incompletely digested vectors during purification of the DNA from the gel, results in a high background of false clones after transformation in E. coli (Figure 1).

The transformation with circular or nicked circular DNA is far more efficient than with linearized DNA. The background can be reduced or even avoided by a preselection (post ligation) digest (2–5), a technique similar to those used in advanced gel-free subcloning methods (6). Here, the final ligation products are digested with an enzyme that does not cut the final plasmid but the religated recipient vector. Finding such an enzyme is in mathematical terms a combinatorial problem. Considering the length of a typical plasmid, which is in the order of several thousands of base pairs, this work, if done by hand, is laborious, time consuming, and highly error prone. Even if programs such as NEBCutter V2.0 (https://nc2.neb.com/NEBcutter2/), Webcutter (http://heimanlab.com/cut2.html), or MacVector (https://macvector.com/) are used, the long lists of cutting enzymes would require further computational processing to identify possible enzymes.

Here, we introduce preselector.uni-jena.de (https://preselector.uni-jena.de/), a freely accessible tool to simplify this tedious combinatorial work. The user can provide up to three sequences, and the tool returns several lists of cutting and non-cutting enzymes for every sequence, as well as a list of enzymes that cut for instance in the donor but not in the final plasmid. Preselector.uni-jena.de is designed so that no sequences are uploaded to the webserver. This does not only speed up the workflow and allows quite long sequences, but also ensures that the sequences remain confidential, compared to other online tools where the sequences are uploaded.

## RESULTS AND DISCUSSION

To close this gap, we have setup the preselector.uni-jena.de interactive web site. There, the user pastes or loads from file two or three DNA sequences into a web form; in the case of a preselection digest, the sequence of the recipient plasmid, the sequence of the final plasmid, and optionally the sequence of the donor plasmid. The sequences can be provided either in plain text or in FASTA format. Additionally, the user may specify whether the input sequences are circular or linear. Finally, preselector.uni-jena.de allows the user to select which enzymes to use from the list of enzymes: By removing a few enzymes from the list, by removing all and putting a few enzymes back, or by loading them from a file.

When the sequences have been entered and the enzymes have been selected, the user hits the button ‘Submit and Digest’ and the site displays several lists of enzymes: One list of enzymes that cut the first but not the third sequence (e.g. the recipient and the final plasmid, respectively), and another list of enzymes that cut the second but not the third sequence (e.g. the donor and the final plasmid, respectively). Additionally, the site displays for each sequence provided a separate list of all cutting and another separate list of all non-cutting enzymes. For each enzyme, the site shows the recognition sequence, the number of matches on the plus and minus strand respectively, and links them to the REBASE website (7) for further information such as methylation sensitivity. We added further details about using our tool in a user guide within the online documentation.

While the purpose for creating preselector.uni-jena.de is to aid the design of preselection digests, it should be noted that it can be more generally used to compare availability of specific restriction enzyme sites between any two DNA sequences. This potentially expands its utility to further applications such as digestion analysis of PCR products, to identify enzymes that may lead to distinctive digestion patterns, something that can be used for genotyping. Another application is finding enzymes for cloning without separating and purifying the DNA fragments on a gel before ligation as Xu *et al.* have suggested (6).

## MATERIALS AND METHODS

The site preselector.uni-jena.de determines whether a set of restriction enzymes cuts in two or three given DNA sequences by searching the DNA sequences for enzyme recognition sites. The restriction enzymes and their recognition sites are loaded from a restriction enzyme database file stored on the server. The file has been derived from REBASE (7) in December 2020 and contains only commercially available enzymes.

The preselection algorithms are implemented in JavaScript, which is downloaded to the local browser and runs there. Therefore, no sequence data is uploaded to the server and all sequences remain confidential. For matching of sequence data, several algorithms are available. The matching of the recognition sites here is implemented with JavaScript's native regular expressions. Before matching, the sequences given by the user via the web interface and the recognition sites of the enzymes are converted to lower case. For each recognition site the reverse complement is generated for matching on the plus and minus strand. And all ambiguous bases in the recognition sites are translated into regular expressions according to the IUPAC notation of the base definitions. For circular matching, the first 30 bp of a sequence are added to its end. After matching, the different lists of enzymes are generated for the user.

## CONCLUSION

With preselector.uni-jena.de, we provide a tool to design all types of preselection strategies: It starts with simple ones, in which we only have to insert a fragment into a multiple cloning site (MCS), and continues with more complex strategies that involve multiple fragments.

Such cases are in the online documentation, where we gave three examples with all the sequences used to illustrate the usefulness of the tool.

In general, preselector.uni-jena.de can be used for any technique in molecular cloning that requires finding enzymes that cut in one sequence, but not in another sequence.

The preselector.uni-jena.de web site provides an easy to use and efficient web interface to design preselection strategies, to make cloning strategies more effective, to potentially avoid costly and time consuming errors, and to solve related combinatorial problems for molecular cloning.

## DATA AVAILABILITY

The preselector site is free to all users. It does not require registration, and is publicly available at https://preselector.uni-jena.de/. The source code is available at https://github.com/MartinGuehmann/preselector/.

## Notes

Present address: Jan Hartung, Institute of Physiology I, Faculty of Medicine, University of Freiburg, Herrmann-Herder-Straße 7, D-79104 Freiburg, Germany.
